# Community occupational therapy for people with dementia and family carers (COTiD-UK) versus treatment as usual (Valuing Active Life in Dementia [VALID] programme): study protocol for a randomised controlled trial

**DOI:** 10.1186/s13063-015-1150-y

**Published:** 2016-02-03

**Authors:** Jennifer Wenborn, Sinéad Hynes, Esme Moniz-Cook, Gail Mountain, Fiona Poland, Michael King, Rumana Omar, Steven Morris, Myrra Vernooij-Dassen, David Challis, Susan Michie, Ian Russell, Catherine Sackley, Maud Graff, Aidan O’Keeffe, Nadia Crellin, Martin Orrell

**Affiliations:** Division of Psychiatry, Faculty of Brain Sciences, University College London, London, UK; Research & Development Department, Goodmayes Hospital, North East London NHS Foundation Trust, Essex, IG3 8XJ UK; Department of Occupational Therapy, National University of Ireland, Galway, Ireland; Faculty of Health and Social Care, University of Hull, Hull, UK; School of Health and Related Research, University of Sheffield, Sheffield, UK; School of Health Sciences, University of East Anglia, Norwich, UK; Priment Clinical Trials Unit, Division of Psychiatry, Faculty of Brain Sciences, University College London, London, UK; Department of Statistical Science and Priment Clinical Trials Unit, University College London, London, UK; Department of Applied Health Research, University College London, London, UK; Department of IQ Healthcare; Kalorama Foundation, Radboud University Medical Center (Radboudumc), Nijmegen, The Netherlands; PSSRU, Faculty of Medical and Human Sciences, University of Manchester, Manchester, UK; Department of Clinical, Educational and Health Psychology, University College London, London, UK; Swansea Trials Unit, College of Medicine, Swansea University, Swansea, UK; Division of Health and Social Care Research, King’s College, London, UK; Department of Rehabilitation and Scientific Institute for Quality of Healthcare-Radboudumc Alzheimer Centre, Radboud University Medical Center (Radboudumc), Donders Institute for Cognition, Brain and Behaviour, Nijmegen, The Netherlands; Institute of Mental Health, University of Nottingham, Nottingham, UK

**Keywords:** Occupational therapy, Dementia, Caregiver, Community, Psychosocial, Activities of daily living, Social participation, Quality of life, Cost-effectiveness

## Abstract

**Background:**

A community-based occupational therapy intervention for people with mild to moderate dementia and their family carers (Community Occupational Therapy in Dementia (COTiD)) was found clinically and cost effective in the Netherlands but not in Germany. This highlights the need to adapt and implement complex interventions to specific national contexts. The current trial aims to evaluate the United Kingdom-adapted occupational therapy intervention for people with mild to moderate dementia and their family carers living in the community (COTiD-UK) compared with treatment as usual.

**Methods/Design:**

This study is a multi-centre, parallel-group, pragmatic randomised trial with internal pilot. We aim to allocate 480 pairs, with each pair comprising a person with mild to moderate dementia and a family carer, who provides at least 4 hours of practical support per week, at random between COTiD-UK and treatment as usual. We shall assess participants at baseline, 12 and 26 weeks, and by telephone at 52 and 78 weeks (first 40 % of recruits only) after randomisation. The primary outcome measure is the Bristol Activities of Daily Living Scale (BADLS) at 26 weeks. Secondary outcome measures will include quality of life, mood, and resource use. To assess intervention delivery, and client experience, we shall collect qualitative data via audio recordings of COTiD-UK sessions and conduct semi-structured interviews with pairs and occupational therapists.

**Discussion:**

COTiD-UK is an evidence-based person-centred intervention that reflects the current priority to enable people with dementia to remain in their own homes by improving their capabilities whilst reducing carer burden. If COTiD-UK is clinically and cost effective, this has major implications for the future delivery of dementia services across the UK.

**Trial registration:**

Current Controlled Trials ISRCTN10748953

Date of registration: 18 September 2014.

## Background

The G8 Summit on Dementia in 2013 pledged to improve life and care for people with dementia and their carers, prevent and delay dementia, and facilitate social adaption to global ageing and dementia [1]. There are 850,000 people living with dementia in the United Kingdom, of whom two-thirds live in the community [2]. Dementia currently costs the UK £26 billion (bn) per year, including £11 bn of unpaid support provided by 670,000 family members and friends. Government policy emphasises the need for high quality, evidence-based interventions [3–6].

Personalised interventions can improve the well-being of family carers, delay admission to care homes, and reduce the risk of institutionalisation by up to one-third [7, 8]. Despite little robust evidence, the NICE-SCIE practice guideline for supporting people with dementia and their carers recommends advice and skills training from an occupational therapist to help those affected to maintain independence [9]. In the Netherlands, Graff et al. [10] developed a clinical guideline for Community Occupational Therapy in Dementia (COTiD) in which they recommended 10 one-hour sessions of home-based occupational therapy over 5 weeks in partnership with the person who has mild to moderate dementia and their family carer to improve skills in the activities of daily living (ADL) and the carer’s abilities and sense of competence. A randomised controlled trial (RCT) of COTiD versus treatment as usual demonstrated improved ADL skills, quality of life, mood and health status, and decreased the need for assistance for the people with dementia; improved sense of competence, quality of life, mood, and health status for their carers [11, 12]; and improved the cost effectiveness [13]. However a subsequent RCT in Germany found no difference between providing COTiD or a single consultation potentially due to the lack of cultural adaptation of the intervention [14]. This highlights the need to adapt complex interventions to specific national contexts [15]. Although COTiD appears to have potential for wider implementation, we therefore need to translate and make it suitable for the UK before evaluating it in a RCT within the Valuing Active Life in Dementia (VALID) research programme.

VALID aims to adapt, develop, evaluate, and implement an occupation-based intervention to improve independence, meaningful activity, and quality of life for people with mild to moderate dementia and their family carers living in the community. The programme structure is based on the Medical Research Council’s (MRC) *‘Framework for development and evaluation of RCTs for complex interventions to improve health’* [16], which describes four phases: development, feasibility and piloting, evaluation, and implementation. The VALID development phase sought to develop the COTiD-UK intervention for subsequent evaluation in an RCT. Activities included translating the Dutch materials; training 44 occupational therapists, who put COTiD-UK into practice with 130 pairs; running focus groups and a consensus event; and conducting a national on-line survey to scope current UK occupational therapy practice and service provision for people with mild to moderate dementia and their family carers living in the community.

This RCT aims to estimate the clinical and cost-effectiveness of the COTiD-UK intervention in comparison with treatment as usual (TAU).

**Fig. 1:**
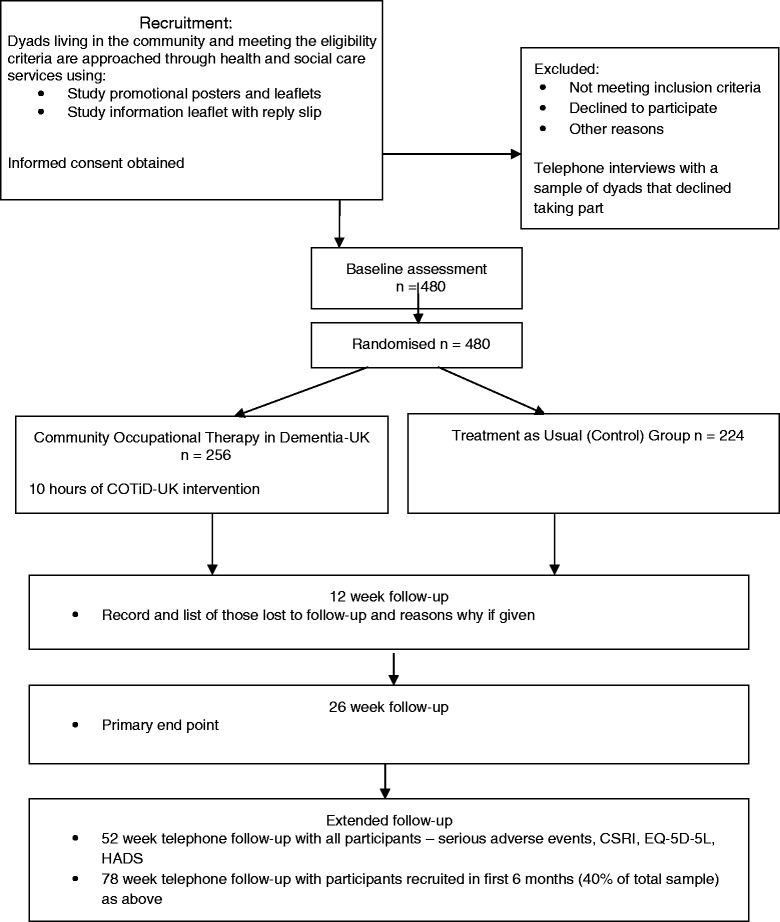
VALID trial flowchart

A CONSORT-style flowchart of the trial is shown at Fig. 1.

## Methods/Design

### Design

This study is a multi-centre, parallel-group, pragmatic randomised trial with internal pilot to estimate the clinical and cost effectiveness of COTiD-UK relative to treatment as usual (TAU). We shall recruit pairs of people with mild to moderate dementia and an identified family carer. We shall allocate recruited pairs at random to TAU and the COTiD-UK intervention in addition to TAU (which may or may not include occupational therapy provision).

The primary outcome measure is the Bristol Activities of Daily Living Scale (BADLS) [17], with a primary end point of 26 weeks. This is considered the optimal time to assess the benefit of COTID-UK on the BADLS whilst reducing the risk of dropout with longer follow-up.

An internal pilot will precede the full RCT to test the outcome measures and trial procedures and to finalise the modes of COTiD-UK delivery, training, and occupational therapist supervision, with the intention of moving forward into the full trial. We shall recruit and randomise 50 pairs for the pilot and use predefined criteria within the ACCEPT framework [18] to review the pilot trial. If the criteria are met to the satisfaction of the independent Programme Steering Committee, the study will proceed to the full trial, by recruiting and randomising another 430 pairs to give a total of 480 pairs. Protocol modifications as approved by the Sponsor will be communicated as appropriate to the Ethics Committee, sites, and participants.

NHS ethical approval was obtained from the NRES Committee London - Camberwell St Giles (reference number 14/LO/0736).

### Setting

The study will run in twelve NHS sites across England. Participating organisations must routinely provide health or social care services to people with mild to moderate dementia and their family carers; employ occupational therapists who work with this population; protect the time needed for at least two occupational therapists to complete the COTiD-UK training, achieve competency and deliver the intervention to ten pairs each; identify a local Principal Investigator; and obtain support from their local Research & Development department and Clinical Research Network to complete all the research activities described in this protocol.

Participants will primarily be recruited from older adult mental health services, notably memory services, but other avenues may be applicable in some areas, for example, liaison psychiatry services or voluntary organisations. We define a family carer as ‘the primary person who feels responsible for, and provides practical support (personal and/or domestic) to, a person with dementia for a minimum of four hours per week. These need not be the closest family member; they could be an extended family member, a close friend, or neighbour and can live together or separately’.

### Participants

#### Inclusion criteria

Participants with dementia must live in their own home (including sheltered accommodation but not a care home), have a diagnosis of dementia as defined by the DSM-IV [19] and score between 0.5 and 2 on the Clinical Dementia Rating Scale [20], be able to converse in English, be able and willing to participate in the COTiD-UK intervention in partnership with their carer, and have the capacity to provide consent. Carers must be aged 18 or over, provide practical support with domestic and/or personal activities to the person with dementia for a minimum of 4 hours per week; and fulfil analogous criteria. Pairs will not be eligible if either is participating in another intervention study.

Occupational therapist participants will be registered as an occupational therapist with the UK Health and Care Professions Council and have experience working in the community or with people who have dementia and their family carers.

### Recruitment

#### Sites and occupational therapists

Recruitment of sites will be via professional and research networks, including the UK National Institute for Health Research (NIHR) Clinical Research Network. Interested organisations will complete a site feasibility checklist to confirm that they meet the inclusion criteria and that adequate resources are available to complete the study in that site. Participating sites will then recruit occupational therapists who meet the inclusion criteria defined above.

#### People with dementia and their carers

We shall brief the clinical staff within the participating services about the study and provide recruitment posters and initial invitation leaflets for potential participants. Research staff will follow a defined screening process to check eligibility, provide the Participant Information Sheet, and complete the recruitment process. The trial recruitment documents were prepared in consultation with people who have dementia and carers in order to maximise accessibility. All recruitment documents and processes are designed to ensure that those with cognitive impairment are fully informed using appropriate language and materials and are engaged in the decision to take part. Strategies include the language and format used in documents, research staff meeting people face to face rather than telephoning, and allowing the time needed to explain the trial to potential participants in order to enable informed choice. It is also important that all research staff members understand the needs of, and are trained to communicate effectively with, this population. All trial participants will receive the VALID Newsletter published every 6 months to keep them informed of the study.

#### Consent process

Informed consent will be obtained from all participants. As people with mild to moderate dementia will be invited to participate, it is anticipated that they will have capacity to understand the implications of taking part and so be able to provide their informed consent, provided sufficient time is taken to explain and appropriate methods of communication are used. The British Psychological Society guidance on evaluating capacity to consent [21] will be followed. As such, obtaining consent is seen as a continuing process, not a one-off decision. It is possible that a person with dementia will lose capacity during the course of the study. If this occurs, then the provisions of the Mental Capacity Act and the associated guidance on nominating a Consultee [22] will be followed. The family carer who is participating in the trial cannot act as the Consultee.

To assess generalisability, each site will maintain a log of people who satisfy the entry criteria for the trial but are not recruited, including basic demographic and clinical details and reasons for not consenting to participate if known.

### Interventions

#### Community Occupational Therapy in Dementia (COTiD-UK)

This manualised intervention consists of up to 10 hours of community occupational therapy for the person with dementia and his or her defined carer. Each pair works in partnership with an occupational therapist to identify meaningful activities and to set appropriate intervention goals. In the first phase, the occupational therapist collects information about both parties and the environment within which the person with dementia lives, including a narrative interview of approximately 1 hour with each individual and observation of the environment and of the person with dementia completing an activity to observe the strategies used by the person with dementia and the carer’s coping style. The occupational therapist facilitates a discussion to agree and prioritise a list of goals to be addressed during the intervention phase. Goals may be joint or individual to either party and can be suggested by any of the three parties. The pair, supported by the occupational therapist, then works through the agreed goals during the intervention phase to enhance the ability of the person with dementia to carry out every day and valued activities. The occupational therapist also coaches the carer to improve their own problem-solving skills and coping strategies to enable the person with dementia to carry out activities and minimise their care burden, thereby better meeting their own needs. The sessions usually take place where the person with dementia lives but, depending on the activities chosen, may also happen in the local community, for example, the sports club, cinema, or garden centre. The intervention typically takes up to 10 hours over 5 to 10 weeks.

### Treatment as usual

The control group will receive standard clinical care but not COTiD-UK or any occupational therapy beyond what they would ordinarily receive. Because the services available to people with dementia and their carers vary between and within sites, each participating site will complete a checklist to describe what they usually provide.

Each participating OT will complete the OT Diary Tool [23] before starting to provide COTiD-UK to describe a ‘typical’ week and for one week whilst providing COTiD-UK. This data will enable comparison of what is provided as usual treatment versus COTiD-UK.

### Fidelity assessment

Fidelity checks to assess how well the delivered COTiD-UK programme adheres to the intervention manual cover five domains: study design, provider training, intervention delivery, intervention receipt, and intervention enactment [24, 25]. Strategies to maximise intervention fidelity include a standardised training programme provided by a consistent team of trainers to the occupational therapists and those supervising them locally and a manual outlining the COTiD-UK ‘active ingredients’ and core intervention materials. Occupational therapists will complete a COTiD-UK Checklist to quantify the number, frequency, length, and content of the sessions provided to each pair. Feedback will be provided by the trainers to support skill development, and regular supervision will be provided by a local COTiD-UK supervisor. We shall audio record COTiD-UK sessions and transcribe a sample to monitor the occupational therapists’ adherence to the intervention, using a checklist derived from the original study.

### Outcome measures

**Fig. 2:**
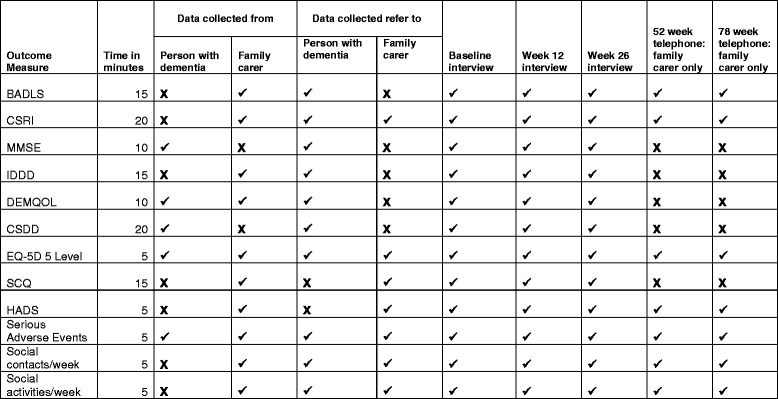
Schedule of outcome measures and assessment points. BADLS, Bristol Activities of Daily Living Scale; CSRI, Client Service Receipt Inventory; MMSE, Mini Mental State Examination; IDDD, Interview of Deterioration in Daily activities in Dementia; DEMQOL, Dementia Quality of Life scale; CSDD, Cornell Scale for Depression in Dementia; EQ-5D-5 L, European Quality of Life scale – 5 Dimensions 5 Levels; SCQ, Sense of Competence Questionnaire; HADS, Hospital Anxiety and Depression Scale

The schedule of outcome measures and assessment points are provided in Fig. 2.

#### Primary outcome measure

##### Bristol Activities of Daily Living Scale

The Bristol Activities of Daily Living Scale (BADLS) [17] measures ability of the person with dementia to complete personal and instrumental activities of daily living. It is a carer rated scale that comprises 20 items including dressing, bathing, food preparation, and using the telephone. Each item scores between zero and three; a total score of 0 shows independence in all activities. BADLS is valid, reliable, and responsive to change over time [26].

#### Secondary outcome measures for person with dementia

##### Cognition

The Mini Mental State Examination (MMSE) [27] is a widely used measure of cognitive function, which is completed by the person with dementia.

##### Condition-specific quality of life

The Dementia Quality of Life scale (DEMQOL) [28] measures five quality of life domains: health, wellbeing, cognitive functioning, social relationships, and self-concept. The person with dementia completes the 28-item questionnaire, and the carer completes the 31-item proxy version.

##### Activities of daily living

The Interview of Deterioration in Daily activities in Dementia (IDDD) [29] measures the level of assistance required by the person with dementia to complete daily living activities by interviewing the carer.

##### Mood

The Cornell Scale for Depression in Dementia (CSDD) [30] screens for depression in people with dementia by interviewing the person with dementia or carer as appropriate.

#### Secondary outcome measures for carer

##### Sense of competence

Sense of Competence Questionnaire (SCQ) [31] measures the carer’s perspective of his/her own competence to cope with the person with dementia.

##### Mood

The Hospital Anxiety and Depression Scale (HADS) [32] is a widely used measure of anxiety and depression.

#### Other data collected

##### Social contact and social activities

We will use the interviews to estimate the number of social contacts and social activities per week as a measure of social functioning.

##### Serious adverse events

We shall record deaths and adverse events that are life threatening, require or extend hospitalisation, result in disability or incapacity, or are otherwise considered significant, together with safeguarding alerts, and report them in accordance with Good Clinical Practice.

### Economic measures

#### Resource use

The Client Service Receipt Inventory (CSRI) [33] is used extensively in studies of mental health and dementia. This inventory gathers data on accommodation, medication, the use of statutory and voluntary organisation services, and inputs from carers. We shall adapt the CSRI to collect resource data from the carer and thereby estimate the cost of dementia care, including unpaid carer inputs.

#### Self-reported health-related quality of life

The European Quality of Life scale – 5 Dimensions 5 Levels (EQ-5D-5 L) [34] provides a simple descriptive profile and a single utility for health status which NICE and many others use to value health-related quality of life. This scale will be completed by the person with dementia and carer at the baseline and follow-up interviews and by the carer only at the telephone follow-up(s).

### Procedure

#### Data collection

Research staff masked to the participants’ allocated group will interview the participants face to face at the home of the person with dementia – at baseline and 12 and 26 weeks after randomisation. They will also phone all carers at 52 weeks and again at 78 weeks for those recruited in the first 6 months of the trial to follow up an estimated 40 % of the total sample for at least 1 year after the end of the intervention but within the period of funding. These two phone interviews will collect data on serious adverse events and allow completion of the CSRI and carer-rated measures including the BADLS, EQ-5D-5 L, and HADS.

#### Randomisation

We shall use a web-based internet clinical trial randomisation service provided by sealed envelope via the PRIMENT Clinical Trials Unit. This will employ permuted blocks of variable size to stratify participants by site and allocate them between intervention and control in the ratio of 1:0.875 to adjust for clustering by occupational therapist.

An unmasked member of the team at each site will conduct this randomisation. Participants will receive letters specifying their allocated group, reminding them what this entails, and enclosing a COTiD-UK information leaflet for those in the intervention group. The unmasked researcher will also inform the relevant COTiD-UK occupational therapist, who will then contact the pair to book a first visit to start the intervention within 2 of randomisation.

#### Masking

In this ‘single-blind trial’, the research staff collecting outcome data, statisticians, health economists, the Programme Steering Committee, and Data Monitoring and Ethics Committee will be masked to participants’ allocations. However, masking the participants or occupational therapists is not possible. To minimise the risk of bias [35], we shall take several precautions.

1. We shall stress the importance of maintaining masking when training research assistants and occupational therapists and encourage them to raise any queries they may have.
2. We shall ask pairs not to tell visiting research staff whether or not they have seen an occupational therapist.
3. We shall minimise contact between masked researchers and occupational therapists.
4. Masked research staff will not have access to any data that could compromise masking, including specific sections of the web-based database.
5. Masked researchers will record after each assessment to which group they judged the pair belonged to and with what level of confidence.
6. We shall record all unmaskings, including the reason – by participants, researchers, clinical staff or others.
7. We shall test in sensitivity analysis whether and how these implicit and explicit forms of unmasking affected the estimated parameters.

### Data management

We shall enter data into a web-based internet clinical trial data capture system (RedPill) provided by Sealed Envelope via PRIMENT Clinical Trials Unit. Staff entering data will each have an individual identifier to access masked or unmasked sections as appropriate. We shall audit the accuracy of data according to the agreed-upon trial monitoring plan. All personal information will be held securely in accordance with Data Protection legislation.

### Sample size

Using a two-sample test and a significance level of 5 %, 172 participants would be required in each arm to detect a difference between the TAU and COTiD-UK groups of 0.35 standard deviations in mean BADLS scores with 90 % power. Allowing for clustering by OT within the COTiD-UK arm, and assuming an average cluster size of 10 pairs and an intra-cluster correlation (ICC) of 0.015, the size of the intervention arm was inflated to 196. Allowing for 15 % attrition at 6 months (estimated from previous similar studies), and 5 % non-adherence increases the total sample size required to 480 pairs: 256 intervention and 224 control. We estimated the sample size using STATA version 11.

### Qualitative data collection

We shall collect and analyse a range of qualitative data to explore the experience of taking part in COTiD-UK, and the feasibility of implementing it in practice. We shall analyse a purposive sample of transcripts of audio recorded COTiD-UK sessions, stratified by the occupational therapist and type of session. We shall conduct semi-structured interviews exploring the experience of providing COTiD-UK with a purposive sample of participating occupational therapists, stratified by grade, experience and service, and sufficient in number to achieve saturation. We shall develop an indicative topic guide for these interviews, which will be conducted over the telephone and recorded.

Semi-structured interviews will explore the experience of COTiD-UK with a purposive sample of participating pairs soon after they have completed the COTiD-UK. We shall stratify interviewed pairs by site, gender, ethnicity, relationship, and number of COTiD-UK sessions received, and we will recruit enough to achieve saturation. An indicative topic guide will be used for these recorded interviews, which will all be conducted face to face and separately with the person who has dementia and with their carer when feasible.

Brief telephone interviews will be conducted with a sample of carers who declined to take part in the trial. When a potential participant declines, we shall ask for their reason and offer the opportunity of discussing this by phone with a member of the central research team. We will ask the person to sign a reply slip consenting to be contacted for a short telephone interview, which will take no longer than 15 minutes and be audio recorded to enable analysis.

### Statistical analysis

Analysis will follow a defined statistical analysis plan approved by the independent Data Monitoring and Ethics Committee before we access any trial data. We do not plan any interim analysis.

The baseline characteristics will be presented descriptively for individuals with dementia and their carers. Categorical variables shall be reported as counts and percentages and continuous variables as mean/median and standard deviation/interquartile ranges as appropriate. Primary analysis will use the BADLS scores at 26 weeks to compare the COTiD-UK and TAU groups. We shall use a random-effects linear regression model to take account of clustering by occupational therapist in the COTiD-UK arm, adjusting for the baseline BADLS score and trial site. We shall investigate potential bias due to missing data. If we identify predictors of missingness that are related to the outcome, we shall adjust the primary analysis with these predictors.

We shall also use repeated measurements of the BADLS score in the secondary outcome analyses using regression models that account for this additional clustering of measurements within participants. We shall also investigate the effect of occupational therapists’ experience, the length of time they have been delivering the intervention, and the number of COTiD-UK sessions delivered, on BADLS scores using an appropriate random effects regression model. As part of a sensitivity analysis, multiple imputation techniques may be undertaken for the analyses related to the BADLS score. We shall perform a dose–response analysis to investigate whether the volume of occupational therapy contributions in TAU and COTiD-UK arms improve outcome measures. We shall use appropriate regression models accounting for clustering to analyse the secondary outcomes. We shall check the assumptions of normality of residuals required by the random effects models and, if violated, consider appropriate transformations. Results from the secondary analyses will be presented as estimates with 95 % confidence intervals, whereas *P*-values will be presented only for the primary comparison. We shall conduct all secondary outcome analyses by treatment allocated, using available data only with no adjustment made for missing data. We shall examine non-adherence in the COTiD-UK group descriptively, and if appropriate, a complier average causal effect (CACE) shall be estimated. A detailed statistical analysis plan will be prepared nearer the analysis stage (before the data are received for analysis).

#### Economic evaluation

We shall analyse cost-effectiveness over the trial period of 12 months, as we seek to follow all participants for at least 1 year. We shall also construct a decision-analytic model to project cost-effectiveness in the long run, namely the expected lifetime of participants, if the intervention confers significant clinical benefits or cost reductions during the trial period. We shall assess costs primarily from the perspective of the NHS and personal social services, but also consider costs to people with dementia and their carers. Thus, we shall cost the COTiD-UK intervention; NHS resources (including general practitioners, practice and community nurses, hospital inpatient, outpatient and day visits for specialist care, occupational therapy and physiotherapy); personal social services resources (including social workers, nursing homes, homecare, meals on wheels and day care); and informal care (including the time of carers and payments by people with dementia and carers). We shall derive unit costs from standard sources.

The short-run model will estimate cost-effectiveness by the incremental cost per QALY gained and per unit change in BADLS. We shall estimate QALYs from the mortality and quality of life data collected during the trial from EQ-5D-5 L and DEMQOL, including the proxy version. We shall construct utility profiles for people with dementia and carers by linear interpolation and estimate QALYs by the area underneath this profile. We shall use multiple imputation to infer missing data on utilities and resource use. We shall estimate cost-effectiveness by dividing the mean cost difference between COTiD-UK and TAU by the mean difference in outcome (in BADLS scores or QALYs) to yield incremental cost-effectiveness ratios (ICER). We shall use non-parametric bootstrapping to derive confidence intervals for ICERs in the face of data skewness. We shall construct cost-effectiveness acceptability curves and subject the results to deterministic sensitivity analysis.

The lifetime model will project cost-effectiveness by the incremental cost per QALY gained. We shall (1) design a model to characterise health states of people with dementia and costs incurred by them and their carers, (2) populate the model using published literature and routine sources, (3) extrapolate final outcomes in QALYs from the trial, and (4) identify which parameters in the model are the most uncertain drivers of cost-effectiveness. We shall base the model on existing long-term economic models in this field. At this stage, we propose a Markovian model of movement between dementia states every 6 months until all members of the synthetic cohort have died from dementia progression or other causes.

#### Qualitative data analysis

We shall use inductive thematic analysis to analyse all qualitative data including: COTiD-UK session transcripts, post-intervention interviews, and telephone interviews with people who declined to participate in the trial. Two of us will code data and identify themes identified. Analysis will be a rigorous, iterative and recursive process, characterised by continual reading and re-reading of the data until saturation of the constructs is achieved.

## Discussion

UK health and social care policy emphasises the importance of people with dementia receiving an early diagnosis; obtaining information and support services quickly and easily; being supported to live in their own homes; and the development of high-quality, evidence-based interventions. Providing community-based programmes that aim to improve the quality of life for people with dementia and their carers, training and supporting carers, and tailoring interventions to individuals are seen as key aspects of this national policy. COTiD-UK is an evidence-based person-centred intervention that reflects the current UK clinical and research priorities for supporting people with dementia and their carers to remain in their own homes.

High-quality, well-controlled intervention studies are essential in order to develop effective services for people with dementia and their carers. Adapting successful interventions from other countries serves as a good basis for service, but successful implementation also requires consideration of the national culture and service context.

This trial aims to determine the clinical and cost-effectiveness of a community occupational therapy intervention for people with dementia and their carers and the feasibility of implementing it within the UK culture and service context. The development and evaluation of complex interventions such as COTiD-UK therefore lend themselves to a mixed-methods approach as described above.

If COTiD-UK is demonstrated to be clinically and cost effective, it will have significant implications for the future provision of services to people with mild to moderate dementia and their carers across the UK.

### Trial status

This trial is currently recruiting. Recruitment started in September 2014 and is due to continue until the spring of 2016.

## Abbreviations

ADL: activities of daily living: can include anything from self-care (washing and dressing) to work or leisure activities
COTiD: Community Occupational Therapy in Dementia, translated from the Dutch EDOMAH
COTiD-UK: Community Occupational Therapy in Dementia - UK version, as developed in the first phase of the VALID research programme
MRC: Medical Research Council
NELFT: North East London NHS Foundation Trust (Sponsor)
NICE: National Institute for Health and Care Excellence
NIHR: National Institute for Health Research (Funder)
OT: occupational therapy/occupational therapist
PRIMENT: Clinical Trials Unit, University College London
RCT: randomised controlled trial
TAU: treatment as usual
VALID: Valuing Active Life in Dementia
